# Supplementary material for: Artificial Selection Finds New Hypotheses for the Mechanism of Wolbachia-Mediated Dengue Blocking in Mosquitoes
Source: Front Microbiol. 2020 Jul 7;11:1456. doi: 10.3389/fmicb.2020.01456 (PMC7358395; doi:10.3389/fmicb.2020.01456)
Supplement: Supplementary file 1 [file Data_Sheet_1.zip › Table S1.docx]

| **Supplementary Table 1. Primers and probes used** | | | | |
| --- | --- | --- | --- | --- |
| **Target organism** | **Target gene (name)** | **Direction** | **Sequence (5’-3’)** | **Tm** |
| Dengue virus | 3’ untranslated region (UTR) | Fw | AAGGACTAGAGGTTAGAGGAGACCC | 54 |
|  |  | Rv | CGTTCTGTGCCTGGAATGATG | 58 |
|  |  | Probe | FAM-AACAGCATATTGACGCTGGGAGAGACCAGA- BHQ1/3 |  |
